# Remote Ischemic Preconditioning Before Drug-Coated Balloon Implantation can Improve the Long-Term Prognosis of Patients with CAD

**DOI:** 10.31083/j.rcm2504116

**Published:** 2024-03-27

**Authors:** Zhenzhou Zhao, Haosen Yu, Ming Nie, Xuejie Li, Muwei Li

**Affiliations:** ^1^Heart Center of Henan Provincial People’s Hospital, Central China Fuwai Hospital, Central China Fuwai Hospital of Zhengzhou University, Henan Provincial People’s Hospital, 450003 Zhengzhou, Henan, China

**Keywords:** percutaneous coronary intervention, remote ischemic preconditioning, drug-coated balloons

## Abstract

**Background:**

Drug-coated balloons (DCBs) have become increasingly vital to percutaneous coronary intervention, offering many advantages. However, a significant challenge is that many patients are intolerant to the myocardial ischemia caused by DCB dilation. Remote ischemic preconditioning (RIPC) is known to enhance heart’s tolerance to ischemia and hypoxia. This study investigated whether preoperative RIPC could extend the tolerated DCB inflation time and improve the long-term prognosis of patients with coronary artery disease (CAD).

**Methods:**

A total of 653 patients with CAD were recruited and randomized into a RIPC group (n = 323) and a control (n = 330) group. The RIPC group underwent RIPC on the left upper limb twice daily, starting three days before the DCB implantation. The patients were followed up for one year after the operation, and 197 patients returned for coronary angiography (CAG) examination where the quantitative flow ratio (QFR) of the target vessels was measured. The primary endpoint of the study was the incidence of target lesion failure (TLF), which included target lesion revascularization (TLR), target vessel myocardial infarction, and cardiac death. The secondary endpoint was the rate of QFR loss in the target vessels.

**Results:**

The findings revealed a significantly lower incidence of TLR in the RIPC group compared to the control group. Additionally, at the one-year follow-up, the rate of QFR loss in target vessels was lower in the RIPC group than in the control group.

**Conclusions:**

The preoperative application of RIPC effectively extended the duration patients could tolerate DCB inflation. Furthermore, this approach positively impacted the long-term prognosis of CAD patients undergoing DCB treatment.

**Clinical Trial Registration Information:**

NCT04766749.

## 1. Introduction

Drug-coated balloons (DCBs) are an emerging technique for treating percutaneous coronary intervention (PCI). They directly release the drug paclitaxel from the balloon surface, swiftly and uniformly, to targeted lesions during balloon inflation [1]. This process effectively inhibits the proliferation and migration of vascular smooth muscle cells, thereby reducing neointimal hyperplasia after angioplasty [1]. Unlike stents, DCBs maintain the coronary artery’s natural anatomy, minimizing vascular wall stimulation and intimal inflammatory responses. While the efficacy and safety of DCBs has been established for treating in-stent restenosis [2, 3, 4] and small vessel disease [5, 6, 7], but their application to large vessel *in-situ* lesions is limited. This limitation arises due to the extensive territories of large blood vessels and the potential for significant myocardial ischemia caused by DCB inflation in these areas.

Remote ischemic preconditioning (RIPC) involves inducing transient, controlled ischemic-hypoxic events in a distant organ (e.g., limb), to reduce the risk of a secondary ischemia/reperfusion injury in a primary target organ following acute ischemia. Approximately 30 years ago, Przyklenk *et al*. [8] described this method discovered in a canine heart model. They demonstrated that inducing non-invasive ischemia in one area of the coronary artery (specifically, the circumflex branch) could protect an adjacent coronary artery from the effects of a prolonged occlusion [8]. This groundbreaking study laid the groundwork for the concept of RIPC [8]. Building on this, Kharbanda *et al*. [9] extended these findings to human subjects, demonstrating the feasibility of inter-organ RIPC. Over time, this approach has been progressively integrated into the clinical management of coronary artery disease (CAD).

The initial CONDI-1 trial (Effect of Remote Ischemic Conditioning on Clinical Outcomes in ST-Elevation Myocardial Infarction) suggested that using RIPC as an adjunct to primary PCI (PPCI)—the gold standard therapy for ST-elevation myocardial infarction (STEMI)—improves myocardial salvage index and left ventricular systolic function after 30 days in patients at risk for massive myocardial infarction [10, 11]. Complementing these findings, the RIC-STEMI trial (Remote ischaemic conditioning in ST-elevation myocardial infarction as adjuvant to primary angioplasty) demonstrated that RIPC further reduces hospitalizations, cardiac deaths, and improves the overall mean ejection fraction at one year, aligning with the outcomes observed in the CONDI-1 study [12].

Several studies have revealed a potential mechanism for the cardioprotective effects of RIPC. For example, RIPC in mice hindlimbs has been shown to increases anti-inflammatory interleukin-10 (IL-10) protein levels in plasma and the heart, inducing Akt activation of Akt and endothelial nitric oxide synthase in the heart, contributing to cardioprotection [13]. Breivik *et al*. [14] reported that coronary ischemic preconditioning effluent from mouse hearts contains potent cytoprotective mediators that protect the myocardium during ischemia-reperfusion through phosphatidylinositol 3-kinase/protein kinase B (PI3K/Akt)-dependent signaling pathways. Additionally, clinical studies have shown that RIPC significantly enhances coronary microcirculatory function and reduces microcirculatory obstruction in patients with STEMI [15, 16]. Although RIPC has demonstrated effectiveness in cases treated with drug-eluting stents, its application prior to DCB treatments remains under-explored.

Currently, the fractional flow reserve (FFR) is recognized as the gold standard for assessing cardiac function [4]. However, due to its invasiveness, complex operation, and high cost, its clinical application is limited. In contrast, the quantitative flow ratio (QFR) emerges as a novel method for evaluating coronary flow reserve. This technique leverages three-dimensional angiographic reconstruction of the target vessel and calculation of intravascular FFR using a fluid dynamics algorithm to assess the functional significance of coronary stenosis [17, 18]. Notably, the QFR has demonstrated commendable accuracy in identifying coronary artery stenosis [19], showing comparable results between online and offline QFR analysis [20].

This study aimed to assess the potential benefits of preoperative RIPC in the context of DCB procedures. Specifically, we aimed to determine if RIPC can extend the duration for which patients can tolerate DCB inflation, a crucial factor in the effectiveness of percutaneous coronary interventions. Additionally, we sought to assess the impact of RIPC, administered prior to DCB treatment, on the long-term prognosis of patients. To achieve this, we employed QFR analysis as a tool for evaluating coronary flow reserve and functional significance of coronary stenosis. By integrating these methodologies, our study aimed to provide new insights into the potential synergistic effects of RIPC and DCB treatments, potentially offering a novel approach to improve clinical outcomes for patients undergoing cardiac interventions.

## 2. Methods

### 2.1 Patients

This study enrolled 653 CAD patients who attended our hospital between January 2020 and January 2022. The inclusion criteria were as follows: preoperative angiography consistent with coronary atherosclerotic heart disease; the expectation of DCB treatment for the lesion; and age >18 years. Exclusion criteria were the following: (1) age >80 years; (2) target vessel occlusion, excessive distortion, or severe calcification; (3) target vessel unsuitable for balloon inflation or direct stent implantation; (4) forward blood flow <thrombolysis in myocardial infarction (TIMI) grade 3 after balloon preconditioning or postoperative residual stenosis of 30%; (5) poorly controlled heart rate (heart rate >100 beats/min) or poorly controlled blood pressure (systolic blood pressure >180 mmHg or diastolic blood pressure >120 mmHg); (6) valvular heart disease, or congenital heart disease accompanied by cardiac insufficiency and severe arrhythmia; (7) previous or current history of severe limb trauma, deep vein thrombosis or thrombophlebitis; (8) severe peptic ulcers, coagulation disorders, history of cranial surgery, active bleeding within the past 6 months; (9) poor general condition, such as severe lung infection, Hepatic and renal insufficiency, malignant tumor; (10) history of allergy to contrast media or drugs; and (11) contraindications to clopidogrel, aspirin, heparin, or paclitaxel.

**Fig. 1.**
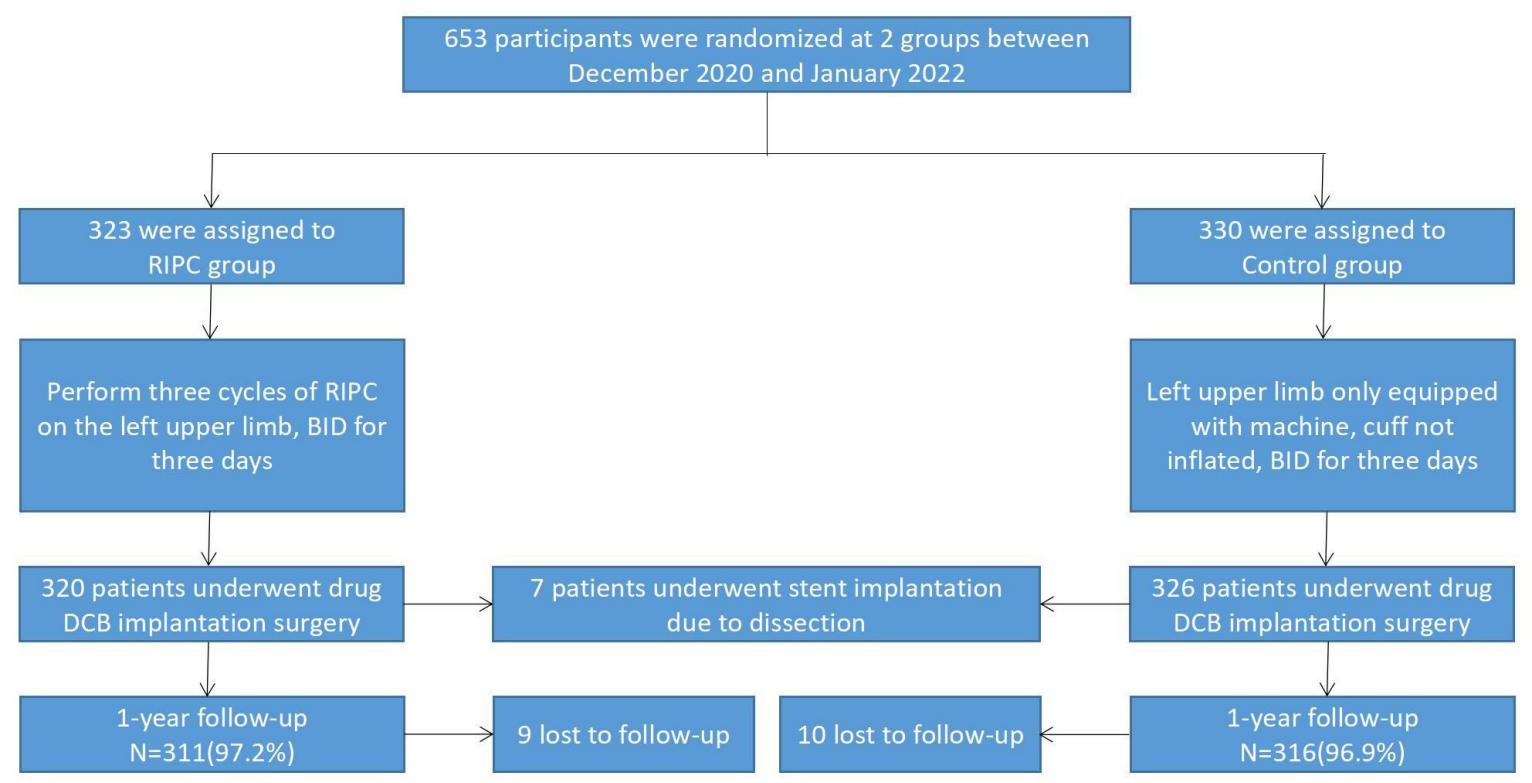
**Study flow chart**. RIPC, remote ischemic preconditioning; BID, bis in die, or twice a day; DCB, drug-coated balloon.

The patients were randomly divided into either the RIPC (n = 323) or the control group (n = 330) before surgery. In the RIPC group, a cuff was inflated over the left upper limb to 200 mmHg three days before the DCB operation. The inflation was maintained to a level where the pulse in the radial and ulnar arteries was no longer palpable, effectively interrupting the distal blood flow. This pressure was sustained for 5 min, after which it was released to allow blood flow to resume. After 5 min of rest, the pressure was reapplied. This process was repeated for three cycles, each lasting a total of 30 minutes, and conducted twice daily over a period of 3 days. Subsequently, routine DCB implantation was carried out. In contrast, patients in the control group underwent a sham procedure where a cuff was placed on the left upper limb but without any inflation. DCB implantation was performed three days later. The flow chart is shown in Fig. 1.

This study was conducted in accordance with the ethical principles of the 1975 Declaration of Helsinki and was approved in advance by the Ethics Committee of Central China Fuwai Hospital. All participants offered written informed consent.

### 2.2 PCI Procedure

This study used Sequent ® Please (Braun, Melsungen, Germany) and Swide ® DCB (Shenqi Medical, Pudong, Shanghai, China) paclitaxel-coated balloons. The DCB for CAD utilization strategy references the German Consensus Group [21]. Treatment with DCB was carried out by more than two experienced interventionalists after optimizing lesion preparation. A standard, semi-compliant balloon was dilated at the target lesion. If the semi-compliant balloon dilatation was unsatisfactory, a high-pressure non-compliant balloon or a cutting and nicking balloon was used. If the target vessel met the diameter stenosis <30% by visual assessment, TIMI flow grade 3, and QFR >0.9, a DCB that exceeded the edge of the lesion by at least 2 mm was used for release at the lesion. The DCB diameter was the same as that of the reference vessel, and the ratio of the balloon to the vessel was 0.8–1.0. If the coronary artery occlusion was greater than or equal to type C, resulting in low lumen benefit, rescue stent implantation was considered.

The duration of DCB inflation was determined and recorded by the interventionalist based on the patient’s clinical presentation and electrocardiographic (ECG) monitoring of ischemic changes. The balloon was deflated when the patient presented with obvious symptoms, ECG abnormalities (such as ventricular tachycardia, ventricular fibrillation, advanced atrioventricular block, and heart rate was <50 beats/min) were observed, or the ST-segment elevation was ≥2.0 mV on ECG.

All patients were treated with dual antiplatelet therapy (DAPT), aspirin (100 mg QD [quaque die, every day]), clopidogrel (75 mg QD), or ticagrelor (90 mg BID [bis in die, twice a day]) according to the 2018 European Society of Cardiology/European Association for Cardio-Thoracic Surgery (ESC/EACTS) Guidelines on myocardial revascularization. Anticoagulation with intravenous heparin was used to maintain an activated clotting time of 250–300 s. After surgery, DAPT was administered for at least 6 months, and aspirin was administered throughout the patient’s life.

### 2.3 QFR

The QFR is a method for rapid analysis of the FFR without a guidewire based on coronary angiography (CAG) images. It combines quantitative CAG and TIMI frame-counting methods. For the single- or double-position (projection angle difference ≥15°) digital subtraction angiography image sequence, optimized coronary artery three-dimensional reconstruction technology, and a fluid mechanics algorithm were used to obtain the blood flow fraction value of the main branch and the branch of the targeted coronary artery segment through simulation calculations. This was used to evaluate the severity of myocardial ischemia in coronary artery stenosis lesions.

Angiographic data were collected from the patients and analyzed using QFR equipment. Through this process we calculated QFR acquisition (defined as postoperative QFR - preoperative QFR), QFR loss (defined as follow-up QFR - postoperative QFR), and target lesion restenosis (defined as follow-up QFR <0.75). The above analysis was performed by three skilled technicians, independently and in a blinded manner. The average values were used as the experimental data.

### 2.4 Follow-up after Operation

All patients underwent clinical treatment or telephone follow-up after the operation. The primary endpoint was the incidence of clinically driven target lesion failure (TLF), which was a combination of cardiac death, target vessel myocardial infarction, and clinically driven target lesion revascularization (TLR). We defined TLR as any repeat revascularization resulting from intra-segmental proximal or distal 50% stenosis treated with DCB. Target vascular thrombosis and bleeding were defined according to Academic Research Consortium guidelines. An independent clinical event committee adjudicated all events.

The secondary endpoint was target lesion restenosis (defined as a follow-up QFR of <0.75). At the end of the one-year follow-up period, 46 patients had experienced TLF. We invited 581 patients who had no TLF events to undergo CAG at our hospital, of whom 197 accepted the invitation. The grouping of the patients did not change (95 were in the RIPC group and 102 were in the control group).

### 2.5 Statistical Analysis

Continuous variables are expressed as mean ± SD or median (interquartile range), and dichotomous variables are expressed as counts and percentages of total. Comparisons of continuous variables were made using the Student’s *t* test and comparisons of categorical variables were made using the Fisher’s exact test, all *p* values were two-tailed and statistical significance was defined as *p* < 0.05. The cumulative event curve was calculated using the Kaplan–Meier method and compared using the log-rank test. The Cox proportional hazards model was used to calculate hazard ratios with 95% confidence intervals. Multivariate Cox regression analysis was performed on factors with a significance level of *p* < 0.2 for the univariate analysis. A mixed-effects model was used to treat lesions from the same patient as random effects. All analyses were performed using SPSS software (version 24.0; IBM Corp., Munich, Germany).

## 3. Results

### 3.1 Demographics and Clinical Baseline Findings

This study enrolled 653 CAD patients who attended our hospital between January 2020 and January 2022 (see Fig. 1 for flowchart). There were no significant differences in age, body mass index, or left ventricular ejection fraction between the RIPC and control groups (*p* > 0.05; Table 1).

**Table 1.** **Baseline clinical characteristics**.

	RIPC (n = 320)	Control (n = 326)	*p*
Age (y)	61.03 ± 10.05	61.72 ± 10.15	0.386
Male	242 (75.6)	254 (77.9)	0.491
Hypertension	196 (61.3)	206 (63.2)	0.611
Diabetes mellitus	102 (31.9)	94 (28.8)	0.401
History of smoking	157 (49.1)	141 (43.3)	0.139
History of drinking	144 (45.0)	126 (38.7)	0.102
Hyperlipidemia	166 (51.9)	147 (45.1)	0.085
ACE-I/ARB	204 (63.7)	185 (56.7)	0.069
β-blockers	196 (61.3)	179 (54.9)	0.102
BMI (${\mathrm{kg/m}}^{2}$)	26.99 ± 4.78	27.12 ± 5.17	0.730
LVEF (%)	49.33 ± 10.35	48.64 ± 9.80	0.382

RIPC, remote ischemic preconditioning; ACE-I, angiotensin converting enzyme inhibitor; ARB, angiotensin II receptor antagonists; BMI, body mass index; LVEF, left ventricle ejection fraction. Values are mean ± SD, median (interquartile range), or % (n).

### 3.2 Comparison of Interventional Surgery Between the Two Groups of Patients

The characteristics of the target vessels, lesion length, reference diameter, length and diameter of the notch balloon, or diameter and length of the DCB did not differ significantly between the RIPC and control groups (*p* > 0.05; Table 2). The DCB expansion time in the RIPC group was significantly longer when compared to the control group (110.91 ± 13.82 s vs. 82.09 ± 22.49 s; *p* < 0.05).

**Table 2.** **Coronary lesion features and interventional procedures**.

		RIPC (n = 320)	Control (n = 326)	*p*
Target vessel				0.643
	LAD/D	161 (50.3)	155 (47.5)	
	LCX/OM	62 (19.4)	61 (18.7)	
	RCA/PDA/PLV	97 (30.3)	110 (33.7)	
Lesion length (mm)		24.47 ± 7.17	25.35 ± 6.08	0.092
Reference vessel diameter (mm)		2.98 ± 0.49	2.93 ± 0.52	0.164
Preoperative QFR		0.22 ± 0.10	0.22 ± 0.11	0.569
Pretreatment balloon		(n = 262)	(n = 273)	
	Diameter (mm)	2.06 ± 0.23	2.03 ± 0.30	0.232
	Length (mm)	17.72 ± 2.77	17.48 ± 2.87	0.323
Scoring balloon		(n = 178)	(n = 183)	
	Diameter (mm)	2.58 ± 0.36	2.57 ± 0.43	0.855
	Length (mm)	13.00 ± 0.00	13.00 ± 0.00	1.000
Cutting balloon		(n = 150)	(n = 149)	
	Diameter (mm)	2.78 ± 0.50	2.70 ± 0.44	0.161
	Length (mm)	9.15 ± 2.24	9.60 ± 2.09	0.078
DCB		(n = 320)	(n = 326)	
	Diameter (mm)	2.75 ± 0.45	2.70 ± 0.48	0.135
	Length (mm)	25.23 ± 7.55	25.44 ± 6.60	0.706
Inflation time (s)		110.91 ± 13.82	82.09 ± 22.49	<0.001
Postoperative QFR		0.94 ± 0.04	0.94 ± 0.04	0.340
QFR acquisition		0.72 ± 0.11	0.71 ± 0.11	0.827

RIPC, remote ischemic preconditioning; LAD/D, left anterior descending/diagonal branch; LCX/OM, left circumflex/obtuse marginal branch; RCA/PDA/PL, right coronary artery/posterior descending artery/posterior lateral; QFR, quantitative flow ratio; DCB, drug-coated balloon. Values are mean ± SD, median (interquartile range), or n (%).

### 3.3 Comparison of 1-year Follow-up of Patients after PCI

**Fig. 2.**
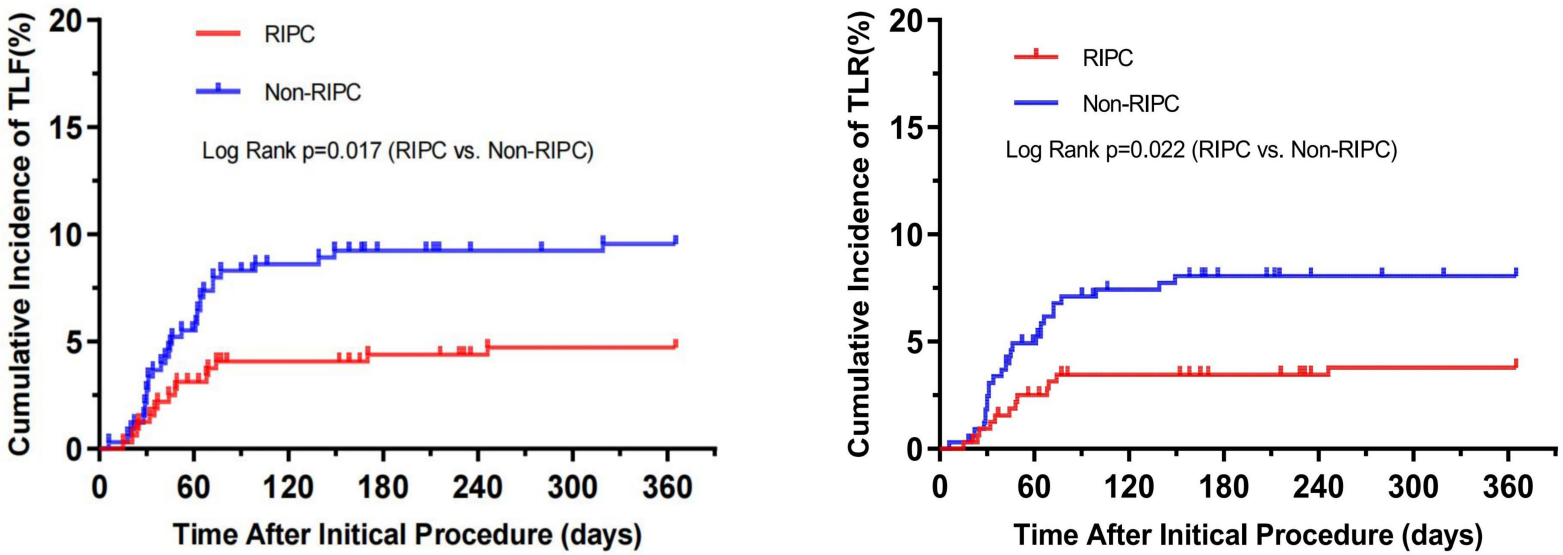
**Cumulative incidence of target lesion failure (TLF) and target lesion revascularization (TLR)**. RIPC, remote ischemic preconditioning.

The incidence of TLF was significantly lower in the RIPC group than in the control group (15 [4.7%] vs. 31 [9.5%]; *p* = 0.017). Similarly, the incidence of TLR was also significantly reduced in the RIPC group when compared to control (12 [3.8%] vs. 26 [8.0%]; *p* = 0.022). However, when considering the incidences of all-cause death, cardiac death, or nonfatal myocardial infarction, no significant differences were observed between the RIPC and control groups (all *p* > 0.05; Table 3). Additionally, Fig. 2 illustrates the cumulative incidence of both TLF and TLR (as estimated by Kaplan–Meier methods) across the two groups, highlighting a statistically significant divergence (TLF: *p* = 0.017, TLR: *p* = 0.022). It’s noteworthy that TLF occurrence is primarily comprised of instances of TLR. This data underscores the potential efficacy of RIPC in reducing specific adverse outcomes following DCB procedures, while not significantly impacting mortality or myocardial infarction rates.

**Table 3.** **Results from the 1-year follow-up following PCI**.

	All	RIPC	Control	*p*
TLF	46 (7.1%)	15 (4.7%)	31 (9.5%)	0.017
Cardiac death	6 (0.9%)	2 (0.6%)	4 (1.2%)	0.409
TVMI	4 (0.6%)	1 (0.3%)	3 (0.9%)	0.315
TLR	38 (5.9%)	12 (3.8%)	26 (8.0%)	0.022
All cause death	12 (1.9%)	4 (1.3%)	8 (2.5%)	0.235

PCI, percutaneous coronary intervention; RIPC, remote ischemic preconditioning; TLF, target lesion failure; TVMI, target vessel myocardial infarction; TVR, target vessel revascularization; Values are n (%).

### 3.4 Comparison of the Angiographic Follow-up of Patients

In this study, we focused on comparing surgical details among the 197 patients who underwent repeat CAG. A key finding was that the duration of DCB expansion in the RIPC group was significantly longer when compared to the control group (110.84 ± 14.12 s vs. 82.75 ± 22.74 s; *p* < 0.05). However, it’s important to note that no other surgical parameters demonstrated significant differences between the two groups. Table 4 shows the intraoperative conditions of these two groups of patients, including the distribution characteristics of the target vessels, lesion length, reference diameter, length and diameter of the notch balloon, and diameter and length of the DCB.

**Table 4.** **Coronary lesion features and interventional procedures related data of patients undergoing re-CAG**.

		RIPC (n = 95)	Control (n = 102)	*p*
Target vessel				0.566
	LAD,D	50 (52.6)	52 (51.0)	
	LCX,OM	25 (26.3)	33 (36.4)	
	RCA,PDA,PL	20 (21.1)	17 (16.7)	
Lesion length (mm)		24.59 ± 7.46	25.13 ± 6.29	0.578
Reference vessel diameter (mm)		2.99 ± 0.49	2.91 ± 0.51	0.293
Pretreatment balloon		(n = 76)	(n = 88)	
	Diameter (mm)	2.05 ± 0.22	2.05 ± 0.25	0.968
	Length (mm)	17.79 ± 2.86	17.52 ± 2.92	0.557
Scoring balloon		(n = 51)	(n = 57)	
	Diameter (mm)	2.58 ± 0.35	2.57 ± 0.44	0.821
	Length (mm)	13.00 ± 0.00	13.00 ± 0.00	1.000
Cutting balloon		(n = 46)	(n = 46)	
	Diameter (mm)	2.76 ± 0.49	2.71 ± 0.42	0.633
	Length (mm)	9.07 ± 2.27	9.67 ± 2.01	0.177
DCB		(n = 95)	(n = 102)	
	Diameter (mm)	2.73 ± 0.45	2.69 ± 0.47	0.484
	Length (mm)	25.56 ± 7.88	25.33 ± 6.69	0.829
Inflation time (s)		110.84 ± 14.12	82.75 ± 22.74	±0.001
Preoperative QFR		0.22 ± 0.11	0.23 ± 0.11	0.534
Postoperative QFR		0.94 ± 0.03	0.94 ± 0.03	0.877
Follow-up QFR		0.90 ± 0.08	0.87 ± 0.14	0.042
QFR acquisition		0.73 ± 0.11	0.72 ± 0.12	0.587
QFR loss		0.04 ± 0.09	0.08 ± 0.14	0.043
Target lesion restenosis		4 (4.2%)	9 (8.8%)	0.193

RIPC, remote ischemic preconditioning; LAD/D, left anterior descending/diagonal branch; LCX/OM, left circumflex/obtuse marginal branch; RCA/PDA/PL, right coronary artery/posterior descending artery/posterior lateral; QFR, quantitative flow ratio; DCB, drug-coated balloon; CAG, coronary angiography. Values are mean ± SD, median (interquartile range), or n (%).

Our results indicate that the QFR of the RIPC group was significantly greater than that the control group (0.90 ± 0.08 vs. 0.87 ± 0.14; *p* = 0.042; Table 4). Additionally, the decrease in QFR, referred to as QFR loss, was significantly lower in the RIPC group than that in the control group (0.04 ± 0.09 vs. 0.08 ± 0.14; *p* = 0.043). This suggests that RIPC may be associated with better maintenance of coronary flow. However, when assessing the rate of target lesion restenosis, no significant difference was observed between the two groups. The restenosis rate was 4.2% (4 patients) in the RIPC group and 8.8% (9 patients) in the control group, with a p-value of 0.193, indicating that the difference was not statistically significant. This result suggests that while RIPC may improve QFR, its impact on reducing the rate of target lesion restenosis is not clearly evident from this data.

### 3.5 Results of Multivariate Cox Regression Analysis

The univariate Cox regression analysis conducted in this study identified several factors with p-values less than 0.2, suggesting their potential relevance in the context of the study. These factors included the characteristics of the target vessel, lesion length, preoperative QFR, and QFR acquisition rates. Given their potential significance indicated by the univariate analysis, these factors were subsequently included in the multivariate Cox regression analysis for a more comprehensive evaluation. The Multivariate Cox regression analysis showed that lesion length emerged as an independent predictor of postoperative long-term TLF events. This finding is detailed in Table 5 of the study. The identification of lesion length as an independent predictor underscores its importance in the prognosis of patients undergoing these procedures and suggests that it could be a key consideration in preoperative assessments and decision-making processes. This insight adds valuable knowledge to the field, potentially guiding future clinical strategies and interventions.

**Table 5.** **Cox regression analysis of TLF**.

	Multivariate	
	Hazard ratio (95% CI)	*p*
RCA	2.450 (0.943–6.368)	0.066
Lesion length (mm)	1.051 (1.005–1.100)	0.031
Preoperative QFR	0.865 (0.000–3408.073)	0.973
QFR acquisition	12.221 (0.004–25722.656)	0.567

RCA, right coronary artery; QFR, quantitative flow ratio; TLF, target lesion failure.

## 4. Discussion

Recent studies have highlighted the increasingly important role of DCBs in the treatment of PCI. Therefore, optimizing perioperative management of DCBs is crucial for enhancing surgical planning and outcomes. Currently, there is no consensus on the impact of prolonged DCB inflation time on patient prognosis, even though the efficacy of drug delivery by DCBs is known to be time-dependent. According to Anderson *et al*. [22], the delivery efficiency of the balloon can reach up to 95% within a 1–4 minute contact period with the vascular wall. However, several factors influence the release efficiency of DCBs, including the release pressure, characteristics of the atherosclerotic plaque, pretreatment techniques [23]. These factors can impede achieving the ideal therapeutic dose, especially when the inflation period is short.

We hypothesized that extending the DCB inflation time could improve patient prognosis under the conditions outlined in the present study. Our findings support this hypothesis, revealing that patients who underwent RIPC could tolerate longer DCB inflation times compared to those in the control group. Additionally, we observed lower rates of TLF and QFR loss in the RIPC group. These results suggest that RIPC may enhance the therapeutic efficacy of DCBs, potentially offering a viable approach to improve patient outcomes in PCI procedures.

### 4.1 Preoperative Short-Term Application of RIPC can Improve the Prognosis of Patients

During the one-year follow-up period of the study, patients in the RIPC group demonstrated notable performance improvements. Despite experiencing longer ischemia times during PCI, these patients exhibited a lower incidence of TLF compared to those in the control group.

The clinical adoption of RIPC has gained considerable attention due to its simplicity, ease of operation, and superior safety profile. Several studies have validated its myocardial protective effects [24, 25, 26], reinforcing the value of this technique in cardiac care. Our previous studies confirmed that RIPC could mitigate cardiomyocyte injury caused by PCI and had the added benefit of prolonging DCB inflation time [27]. This study, with an expanded sample size, supports our previous findings, consistently showing that RIPC effectively extends DCB inflation time. Moreover, the one-year follow-up data, provides compelling evidence that extending the DCB inflation time through prior RIPC improves patient prognosis.

Although RIPC has been used in clinical practice for decades, there is still no consensus on the optimal RIPC strategy. This lack of agreement can be attributed to several factors. One critical aspect is that different cycles and ischemic areas may yield different outcomes, especially among subjects of different racial backgrounds [28]. Additionally, conditions including hyperlipidemia, diabetes, and hypertension, along with their associated therapeutic drugs, can potentially interfere with the efficacy of RIPC [29, 30, 31, 32]. These factors may contribute to the inconsistent results observed in different studies. For instance, the combined CONDI-2/ERIC-PPCI trial (Effect of Remote Ischaemic Conditioning on Clinical Outcomes in ST-elevation Myocardial Infarction Patients Undergoing Primary Percutaneous Coronary Intervention) demonstrated that RIPC did not add any clinical benefit when used during PPCI for STEMI during the one-year postoperative follow-up [33]. However, the RIC-STEMI study reported that RIPC significantly reduced cardiac mortality and hospitalizations due to heart failure, achieving an improved combined hard clinical endpoint in patients with STEMI [12]. To minimize the impact of potential confounding factors, our study implemented strict inclusion and exclusion criteria. We ensured that the demographic characteristics, clinical features, and medication usage were statistically comparable between the RIPC and control groups. his rigorous approach was aimed at isolating the effects of RIPC, thereby providing a clearer understanding of its impact on patient outcomes following PCI. By controlling for these variables, the study aimed to deliver more definitive conclusions about the effectiveness of RIPC in this clinical setting.

### 4.2 Increased DCB Inflation Time Benefits QFR during Target Vessel Follow-up

In our study, angiographic follow-up of patients revealed that the RIPC group had a higher QFR of target vessels (*p* = 0.042) and a lower QFR loss (*p* = 0.043) compared to the control group. It’s important to note, however, that while the number of restenosis cases was lower in the RIPC group than in the control group, the difference in the incidence of restenosis between the two groups was not statistically significant (*p* = 0.193). This lack of significant difference could potentially be attributed to the relatively small sample size of the study, which might limit the statistical power to detect a true difference. Our results indicated that an increase in the inflation time of the DCB improved the long-term blood flow reserve of the patients, resulting in positive lumen remodeling.

The observed findings in this study can be attributed to two key factors. First, during PCI, the use of a nicking balloon to cut the plaque inevitably damages the vascular endothelium, resulting in endothelial denudation and destruction of the endothelial cell layer [34, 35, 36]. This triggers a cascade of events including inflammation, platelet activation, as well as the release of growth factor and pro-inflammatory cytokines [34, 35, 36]. These factors may contribute to phenotypic changes in vascular smooth muscle cells, resulting in intimal hyperplasia, ultimately leading to the formation of restenosis [37, 38, 39].

Second, the preoperative application of RIPC may protect vascular endothelial cells, reduce vascular intimal injury, and prevent vascular endothelial dysfunction after acute inflammatory stimulation [40, 41]. Additionally, prolonging the DCB inflation time may increase the drug concentration in the target vessel wall, ensuring that an ideal therapeutic dose is achieved [42]. Together, these mechanisms may explain the beneficial effects observed in patients who underwent RIPC before PCI.

### 4.3 Study Limitations

These experiments represent an update to our previous study [27], where we confirmed that the preoperative administration of RIPC to patients can prolong the DCB inflation time and reduce intraoperative myocardial damage. That work had been limited by a small sample size [27]. Building on these initial findings, we refined our RIPC strategy, expanded the sample size, and conducted a one-year follow-up to evaluate the surgical outcomes, which proved to be encouraging.

However, our current study is not without its limitations. Being a single-center study, the patient population was geographically concentrated, which might influence the findings. Additionally, the sample size, though larger than in our previous study, remained relatively small and consisted exclusively of patients from a single racial background. This homogeneity in the patient demographic poses a limitation to the broader applicability and generalizability of our results to diverse populations.

## 5. Conclusions

This study investigated the relationship between prolonged DCB inflation time and the long-term prognosis of patients with CAD who underwent RIPC prior to PCI. Our findings revealed that prolonging the DCB inflation time improved the long-term QFR of the target vessel and reduced the incidence of TLR. These results suggest that the short-term preoperative application of RIPC may be an effective strategy to enhance the therapeutic benefits of DCBs in CAD patients.

## Data Availability

The data sets generated and/or analyzed during the current study are not publicly available due to protecting patient privacy but are available from the corresponding author on reasonable request.
